# Keeping or changing? Two different cultural adaptation strategies in the domestic use of home country food plant and herbal ingredients among Albanian and Moroccan migrants in Northwestern Italy

**DOI:** 10.1186/s13002-019-0290-7

**Published:** 2019-02-12

**Authors:** Michele Fontefrancesco, Charles Barstow, Francesca Grazioli, Hillary Lyons, Giulia Mattalia, Mattia Marino, Anne E. McKay, Renata Sõukand, Paolo Corvo, Andrea Pieroni

**Affiliations:** 1 University of Gastronomic Sciences, Piazza Vittorio Emanuele II, 9, I-12060 Pollenzo, Cuneo Italy; 2 Present address: Ark of Taste Office, Slow Food, Via Mendicità Istruita 14, I-12042 Bra, Cuneo Italy; 3 Present address: Bioversity International, Viale Tre Danari 472, I-00054 Maccarese Stazione, Rome Italy; 4 Department of Environmental Sciences, Informatics, and Statistics, Ca’ Foscari University of Venice, Via Torino 155, I-30172 Mestre, Venezia Italy

**Keywords:** Migrants’ food, Ethnobotany, Albanians, Moroccans, Piedmont, Italy

## Abstract

**Background:**

Ethnobotanical field studies concerning migrant groups are crucial for understanding temporal changes of folk plant knowledge as well as for analyzing adaptation processes. Italy still lacks in-depth studies on migrant food habits that also evaluate the ingredients which newcomers use in their domestic culinary and herbal practices.

**Methods:**

Semi-structured and open in-depth interviews were conducted with 104 first- and second-generation migrants belonging to the Albanian and Moroccan communities living in Turin and Bra, NW Italy. The sample included both ethnic groups and genders equally.

**Results:**

While the number of plant ingredients was similar in the two communities (44 plant items among Albanians vs 47 plant items among Moroccans), data diverged remarkably on three trajectories: (a) frequency of quotation (a large majority of the ingredients were frequently or moderately mentioned by Moroccan migrants whereas Albanians rarely mentioned them as still in use in Italy); (b) ways through which the home country plant ingredients were acquired (while most of the ingredients were purchased by Moroccans in local markets and shops, ingredients used by Albanians were for the most part informally “imported” during family visits from Albania); (c) quantitative and qualitative differences in the plant reports mentioned by the two communities, with plant reports recorded in the domestic arena of Moroccans nearly doubling the reports recorded among Albanians and most of the plant ingredients mentioned by Moroccans representing “medicinal foods”.

**Conclusion:**

A large portion of the differences shown by the two communities are linked to different methods of procurement of home country gastronomic botanical ingredients, the different transnational informal exchanges that exist between Italy and migrants’ home countries, the presence of markets and ethnic shops in Italy selling these items, and the different degree of difficulty in accessing public health services. The observed divergences were also clearly related to very diverse adaptation strategies, i.e., processes of negotiating and elaborating Albanian and Moroccan cultural identities.

## Background

The intersection between food and migration studies has been analyzed in recent years under two main perspectives. One trend has followed the concept of *nutritional transition* focusing on changes in the dietary habits of migrants and their implications for health [1–6], while another research path has focused on the adaptation of migrants to new ecological and cultural environments by analyzing their dietary habits or the domestic uses of food and/or healthcare plant ingredients [7–20].

In particular, as they relocate, migrants have several options: they can keep or intensify the use of products from their home countries in an attempt to “preserve” their identity, possibly adapting their methods of procurement and enabling them to continue to use traditional items in the new environment; they can adapt their traditional food and domestic medical systems by replacing their usual ingredients with new ones readily found in their new environment; or they can mix “original” and “new” ingredients, inventing new dishes and creating new folk culinary and/or herbal knowledge [21, 22]. Moreover, these frameworks can dynamically change during the timespan of the migratory process and across different generations (i.e., first versus second or third migrant generations).

Even though Italy has faced a huge shift in its multi-cultural mosaic over the last four decades, no extensive research study has addressed the specific issue of the food ingredients used, changed, or manipulated within the domestic arena of migrants, with only the exception of a few sporadic field studies [23–26].

However, domestic ingredients, scents, and flavors represent not only a framework within which migrants may “keep” or “deny” deep ties with their own home identities, but also an effective means to negotiate these identities [27–32].

This research aimed to explore continuity and change in the domestic use of food plant ingredients among the second and third largest migrant groups of both Italy and the Piedmont Region (NW Italy), namely Moroccans and Albanians [33]. The two groups have moved into Italy in different waves within the last four decades: Moroccans especially during the 1980s and 1990s and Albanians particularly during the 1990s [34, 35].

The fact that the two groups are about equal in size and are by far the most numerous Muslim migrant residents in Italy—Islam is the faith of all Moroccans and approximately half of the Albanians in Italy [36]—makes them an ideal arena for comparatively studying their ethnobotanies, i.e., the domestic food and medicinal plant ingredients used and their changes over time.

Accordingly, the objectives of this study were to document the identity, use, provenience, and frequency of use of “original” food plant ingredients utilized in the domestic sphere of Albanians and Moroccans living in two selected sites in Piedmont (Bra and Turin); to compare them, as well as their frequency of use and quotation and their ways of procurements; and to generally understand how that may be linked to similar or different cultural adaptation strategies.

## Methods

### Historical and anthropological background of the Albanian migration to Italy

Albania is a small mountainous country on the Balkan Peninsula and just a few kilometers separate the Albanian coast from Apulia, SE Italy; this proximity and a long history of interaction have united these countries. While the first ancestors of modern Albanians were the Illyrians, starting in the seventh century BC, Illyrian territories became part of the Roman, Byzantine, and then Ottoman Empires (fifteenth to twentieth centuries). Several Albanians migrated to Italy during the sixteenth and seventeenth centuries, settling in rural areas of Southern Italy, where, known as Arbëreshë, they still live and partially speak the language of their ancestors. Albania obtained its independence from the Ottoman Empire in 1912, but during the Second World War, the country was occupied by Italian Fascists, and then in January 1946, the (Communist) People’s Republic of Albania was proclaimed [37]. The dictator Enver Hoxha remained in power until his death in 1985; Hoxha’s communism initially followed that of the Soviet Union and then the People’s Republic of China until 1978, when Albania became totally isolated from the rest of the world [37]. In 1991, when the Communist period ended, a first massive emigration to Italy occurred. The Albanian migration to Italy generally occurred during four periods in the 1990s [38]:

- the “embassy migrants” period that took place during the summer of 1990, when around 20,000 Albanians left the country mainly for Italy thanks to the liberalization of passport issuing;
- the “main mass exodus” that began in the spring and summer of 1991, when 25,000 Albanians reached Apulia by boat and settled in Italy, and continued for the next 2 years during which around 300,000 Albanians left their home country;
- the migration due to the collapse of an investment pyramid scheme that took place during the spring of 1997;
- the 1999 migration wave that occurred during and after the Kosovo War crisis.

According to a report of Italian National Institute of Statistics, approximately 440,000 Albanians lived in Italy in 2017 (of which around 42,000 resided in Piedmont) and they represent—after Romanians—the second largest migrant community living in the country. Half of them work in the industrial sector [39].

### Historical and anthropological background of the Moroccan migration to Italy

Berbers have been living in Morocco since the second millennium BC; however, in 46 A.D., Morocco was annexed by Rome and became part of the Province of Mauritania; and later, in the fifth century, the Vandals overran the region. Then in 685 A.D., Arabs invaded Morocco bringing Islam to the country [40]. In 1904, France and Spain decided upon the control zones in Morocco, and before the beginning of World War I, the territory of present-day Morocco was colonized. Even as Berber uprising resulted in the abandonment of slavery, Morocco remained a colony and Moroccan soldiers served in the French army during World War II and took part in the Spanish Civil war in 1936. It was only in 1956 that France and Spain recognized the independence and sovereignty of Morocco, and since that time, a king has been governing the country [41, 42].

Since the mid-1970s, migration from Morocco to Europe has been continuous. According to De Bel-Air [43], two main phases can be distinguished: the first when Moroccan workers moved primarily to France, Germany, Belgium, and the Netherlands and the second from the 1980s to the late 2000s, when the number of emigrated Moroccans increased, partly through family reunification. This last wave of low-skilled emigrants settled mainly in Spain and Italy working in the agricultural and construction sectors.

As of January 2017, approximately 420,000 Moroccans live in Italy (of which around 54,000 reside in Piedmont) and they represent—after Romanians and Albanians—the third largest migrant community living in the country [33]. Moroccans in Italy mainly work in industry, trade, and restaurants and have a slightly lower level of education compared to that of Albanian migrants and are therefore employed in less specialized manual labor and earn lower wages [36].

### Field study

**Fig. 1:**
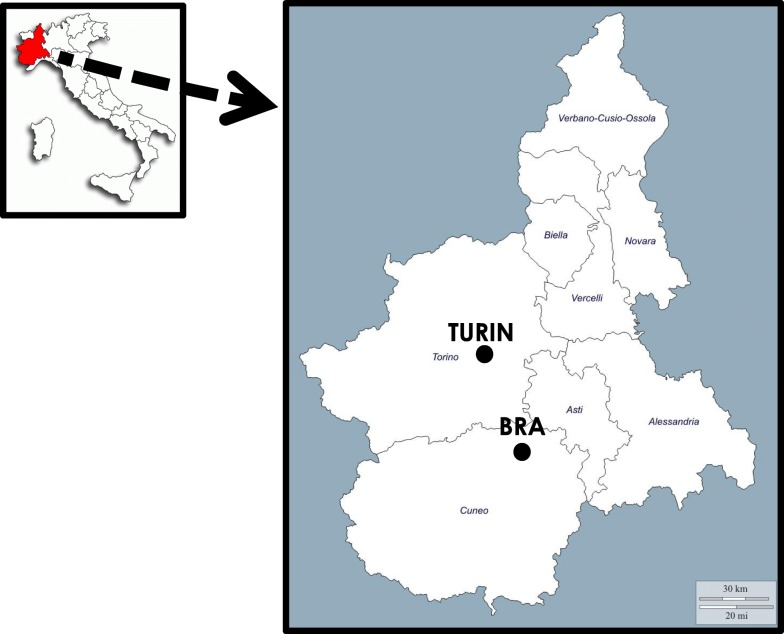
Geographical location of the study sites

The field study was carried out between the spring of 2013 and the beginning of 2017 in the town of Bra, which has about 30,000 inhabitants, and the city of Turin, the capital of the Piedmont Region, which has approximately 900,000 inhabitants (Fig. 1). Semi-structured and open interviews were conducted, by two dozen interviewers having different multi-cultural backgrounds (including one Albanian, one Pakistani, and one Turkish interviewer), with a sample of 54 Albanian and 50 Moroccan first- and second- generation migrants, aged between 18 and 89 years, equally divided between genders (25 men and 29 women among Albanians and 22 men and 28 women among Moroccans), with an average age of 50 among Albanians and 58 among Moroccans. The participants were selected using the snowball sampling technique. All the Moroccans identified as Muslim, while the Albanian sample was half Catholic and half Muslim, although a few Albanians claimed not to be particularly interested in religious affiliation.

The focus of the interviews was the home country food plant and herbal ingredients that were still used in the domestic arena and that had a divergent use from Italian culinary traditions, their ways of procurement, their specific manipulations and detailed uses in the kitchen, and more generally—via open questions—how these ingredients and related dishes “shape” migrants’ identity and sense of belonging. Specific attention was paid to recording “medicinal foods,” i.e., food ingredients consumed with the express purpose of improving general health or preventing or curing specific illnesses. Prior informed consent was obtained before each interview and ethical guidelines of the International Society of Ethnobiology were followed [44]. The very few recorded *wild* plant ingredients were identified following Italian, Moroccan, and Albanian floras, as well as the botanical-ethnolinguistic literature [45–51]. Botanical nomenclature followed The Plant List database [52], while family assignments adhered to the Angiosperm Phylogeny Group standards [53].

Data were analyzed using transcripts of the interviews as well as nutritional anthropological and ethnobotanical literature concerning migrants [1–32].

## Results

### Plant food and herbal ingredients used by the two migrant communities

Table 1 and Table 2 show the plant food and herbal ingredients of the home countries which were still in use within households of the two migrant communities (Albanians and Moroccans, respectively). For each plant ingredient, we reported the recorded folk name, the way in which it was acquired, the parts used, the exact preparation, and the food, medicinal, or medicinal food use as mentioned by the study participants, as well as the quotation frequency.

**Table 1:** Home country food plant and herbal ingredients used by Albanian migrants in Piedmont

Botanical name of the plant ingredient and its botanical family	Common English name	Recorded Albanian name	Locally acquired (L) (cultivated, purchased or gathered from the wild [G]) or informally imported from Albania (I)	Part(s) used	Recorded food (F), medicinal (M), and food-medicinal (F/M) uses	Frequency of quotation
*Abelmoschus esculentus* (L.) Moench, Malvaceae	Okra	Bamja	L	Fr	F: vegetable	++
*Allium cepa* L., Amaryllidaceae	Onion	Qepa	L	Bu	M: crushed and topically applied with garlic and vinegar for treating knee pain	+
*Allium sativum* L., Amaryllidaceae	Garlic	Hudhra	L and I	Bu	F: preserved in oil; seasoning home-made lacto-fermented (salt brined) pickles (*turshi*) M: hang under the stroller as an anti-Evil Eye agent	+++
*Allium ampeloprasum* L., Amaryllidaceae	Leek	Preshi, Purri	L	St	M: topical applications for treating ear-aches	+
*Beta vulgaris* L., Amaranthaceae	Beetroot and chard	Panxhari	L	Le	F: preserved in oil (beet root) and filling for salty pies (*byrek*) (chard)	+
*Brassica oleracea* L., Brassicaceae	Cabbage	Lakra	L	Le	F: *sarma* (leaves rolled around filling of rice and meat)	+
*Capsicum annuum* L., Solanaceae	Chili and bell pepper	Djegës, Speci	L and I	Fr	F: fermented in sour ricotta (*gjizë*) or preserved in oil (chili)	+
*Cichorium intybus* L.*,* Asteraceae	Wild cichory	Rrapiqe	L (G)	Le	F: filling for salty pies (*byrek*)	+
*Citrullus lanatus* (Thunb.) Matsum. and Nakai, Cucurbitaceae	Water melon	Shalqi	L and I (seeds)	Fr	F: consumed raw	+
*Citrus limon* (L.) Osbeck, Rutaceae	Lemon	Limon	L and I	Fr	M: against flu and as an antiseptic	+
*Cucumis melo* L., Cucurbitaceae	Melon	Bostan, Piepen	L and I (seeds)	Fr	F: consumed raw	+
*Cucurbita pepo* L., Cucurbitaceae	Squash	Kungull	L	Fr	F: filling for pies (*byrek*)	+
*Cydonia oblonga* Mill., Rosaceae	Quince tree	Ftoj	L	Wp	M: used for weather forecasting - if it blossoms in May, the following winter will be very cold	+
*Ficus carica* L., Moraceae	Fig	Fik	L and I	Fr	F: jam	+
*Foeniculum vulgare* Mill., Apiaceae	Fennel	Koper	L and I	Fr	F: seasoning cabbage *sarma* M: digestive and anti-bacterial teas	++
*Juglans regia* L., Juglandaceae	Walnut	Arra	L and I	Se	F: *kadaif* and *ashure* sweets	+
*Laurus nobilis* L., Lauraceae	Laurel	Dafën	L and I	Le	M: digestive and expectorant teas; when burnt in a baby’s room, the smoke protects the child against the Evil Eye	++
*Malva sylvestris* L., Malvaceae	Mallow	Mullaga	L (G) and I	Le	M: topically applied to wounds for healing them; drunk as a tea against asthma; as an anti-Evil Eye agent	+
*Matricaria chamomilla* L., Asteraceae	Chamomille	Kamomill	L and I	Ap	M: tea against flu and for calming	+++
*Mentha* spp., Lamiaceae	Mint	Mender, Nenexhik	L and I	Le	M: tea for treating flu	++
*Ocimum basilicum* L., Lamiaceae	Basil	Borzilok	L	Le	M: as an anti-Evil Eye agent	+
*Orchis* spp., Orchidaceae	Wild orchid	Salep	I	Ro	F/M: powdered and boiled in milk; in winter often drunk with a piece of bread	+
*Origanum vulgare* L., Lamiaceae	Oregano	Rigon, Çaj mali	I	Ap	M: tea used recreationally, for treating inflammations, depurative, as a panacea	+++
*Papaver rhoeas* L., Papaveraceae	Corn poppy	Lulëkuqe	I	Fl	M: tea as a calming agent and a hemostatic	+
*Pelargonium* spp., Geraniaceae	Geranium	Lulëbarbarosa	L and I	Le	F/M: syrup, also used as an astringent	+
*Petroselinum crispum* (Mill.) Fuss, Apiaceae	Parsley	Majdanoz	L	Le	F: seasoning home-made pickles (*turshi*)	++
*Phaseolus vulgaris* L., Fabaceae	Green beens	Bishtaja	L and I (seeds)	Fr	F: vegetable	+
*Prunus avium* (L.) L., Rosaceae	Cherry	Qershia	I	Fr	F: home-made alcoholic macerate	+
*Prunus dulcis* (Mill.) D.A.Webb, Rosaceae	Almond	Bajame	L and I	Se	F: *kabuni* sweet	+
*Punica granatum* L., Lythraceae	Pomegranate	Shegë	L and I	Fr	F: pulp processed in juice; M: fruit epicarp decocted and used externally for treating skin problems	+
*Rosa canina* L., Rosaceae	Rose hip	Dranofile i egër	I	Fr	F: jam	+
*Rubus ulmifolius* Schott, Rosaceae	Blueberry	Ferra	L (G)	Fr	M: tea as a depurative	+
*Rumex* spp., Polygonaceae	Sorrell	Lepjet	L (G)	Le	F: filling for salty pies (*byrek*)	+
*Salvia officinalis* L., Lamiaceae	Sage	Sherbela	L and I	Le	F: seasoning; M: digestive tea	+
*Silene vulgaris* (Mill.) Garke, Caryophyllaceae	Bladder campion	Vesh lepri	G	Le	F: filling for salty pies (*byrek*)	+
*Solanum lycopersicum* L., Solanaceae	Tomato	Domate	L	Uf	F: pickled	+
*Solanum tuberosum* L., Solanaceae	Potato	Patate	L	Tu	F/M: soup for treating flu	+
*Solanum melongena* L., Solanaceae	Aubergine	Patëllxhan	L	Fr	F: pickled, roasted	++
*Spinacia oleracea* L., Amaranthaceae	Spinach	Spinaq	L	Le	F: filling for salty pies (*byrek*)	+
*Syderitis* spp., Lamiaceae	Mountain tea	Çaj mali	I	Ap	M: tea used recreationally, for treating inflammations, depurative, as a panacea	+++
*Trigonella foenum-graecum* L., Fabaceae	Fenugreek	Trëndelinë	L and I	Se	F: seasoning; M: as a lucky charm	+
*Urtica dioica* L., Urticaceae	Nettle	Hithër	L (G)	Le	F: filling for pies (*byrek*); M: teas drunk for treating rheumatisms or externally applied as a means for strengthening hair	++
*Vitis labrusca* L., Vitaceae	Fox grape	Rrush çelek	I	Fr	F: consumed raw	+
*Ziziphus jujuba* Mill., Rhamnaceae	Giuggiolo	Kymçe	I	Fr	F: consumed raw/fermented	+

*Ap* aerial parts, *Bu* bulbs, *Fl* flowers, *Fr* fruits, *Le* leaves, *Ro* roots, *Se* seeds, *St* stems, *Tu* tubers, *Uf* unripe fruits, *Wp* whole plant

+, mentioned by less than 10% of the study participants; ++, mentioned by 10–39% of the study participants; +++, mentioned by at least 40% of the study participants

**Table 2:** Home country food plant and herbal ingredients used by Moroccan migrants in Piedmont

Botanical name of the plant ingredient and its botanical family	Common English name	Recorded Arabic name	Locally acquired (L) (cultivated, purchased or gathered from the wild [G]) or informally imported from Morocco (I)	Part(s) used	Recorded food (F), medicinal (M), and food-medicinal (F/M) uses	Frequency of quotation
*Allium sativum* L., Amaryllidaceae	Garlic	Thawm, Tuma	L	Bu	F/M: consumed for treating cold and flu and as an anti-bacterial agent (also macerated in oil for 1 month and then oil used when needed)	++
*Aloysia citriodora* Palau, Verbenaceae	Lemon verbena	Luisa	L	Le	M: tea for treating digestive discomforts, headaches, fever, and as a relaxing agent	++
*Argania spinosa* (L.) Skeels, Sapotaceae	Argan	Argan	L and I	Fr- > Oil	F: garnishing cous cous; dip for flatbread; used to prepare *amlou*, a cream made with argan oil, almonds, and honey; M: externally applied for treating dermatitis and dry skin and on the hair (cosmetic); internally as a cholesterol reducer	++
*Artemisia arborescens* (Vaill.) L., Asteraceae	Wormwood	Sheeba	L and I	Le	M: tea (alone or added to mint tea), for treating cough	+++
*Capsicum annuum* L., Solanaceae	Chili	Filfil harr	L	Fr	F/M: seasoning, considered “good for blood circulation”	++
*Cinnamomum verum* J.Presl, Lauraceae	Cinnamon	Karfa	L	Ba	F/M: spice for savory and sweet dishes; considered able to counteract diabetes and to relief menstrual pains	++
*Citrus limon* (L.) Osbeck, Rutaceae	Lemon	Liymun	L	Fr	F: pickled in brine M: ingredients for treating various diseases (mixed with turmeric, ginger, epazote, and sage)	++
*Cladanthus mixtus* (L.) Chevall. and *Matricaria chamomilla* L.*,* Asteraceae	Moroccan and Italian chamomile	Babunj	L and I		M: tea used for treating stomach-aches and menstrual cramps, and as a calming agent; given to sick children	++
*Coriandrum sativum* L., Apiaceae	Coriander	Kusbar	L	Fr	F: cooking spice for savory dishes; universal spice mix base	++
*Crocus sativus* L., Iridaceae	Saffron	Zaafaran al hur	L	Sg	F: cooking spice and colorant for savory and sweet dishes; F/M: consumed for treating stomach discomforts and fever during pregnancy	++
*Cucurbita pepo* L., Cucurbitaceae	Squash	Zeret gara	L	Se	F/M: consumed for prostate health	+
*Cuminum cyminum* L., Apiaceae	Cumin	Kamun	L	Fr	F: cooking spice for lentil and fish dishes; F/M: consumed as a means for treating stomach diseases, liver infections and as a digestive (also fruits macerated in water and then drunk)	+++
*Curcuma longa* L., Zingiberaceae	Turmeric	Karkoum	L and I	Ro	F: seasoning and colorant for savory dishes; F/M: consumed, it is considered able to treat colds and skin and liver diseases, and as a panacea (sometimes mixed with lemon juice and honey)	+++
*Dysphania ambrosioides* (L.) Mosyakin and Clemants, Amaranthaceae	Epazote	Mkhinza	I	Le	M: tea as an anti-fever agent (sometimes with lemon juice); also mixed with red onion and Bible hyssop to create a paste to be applied to the head	++
*Elettaria cardamomum* (L.) Maton, Zingiberaceae	Cardamom (green)	Huba alhal	L	Fr	F: cooking spice for savory dishes	+
*Ficus carica* L., Moraceae	Fig	Shreha	L	Fr	M: fruits left in olive oil for approx. One month, then oil drunk as needed for treating constipation	+
*Foeniculum vulgare* Mill., Apiaceae	Fennel	Alshamra	L	Fr	F: garnish for sweets and breads; cooking spice for savory dishes; M: tea considered good as a digestive (sometimes ground fruit with coffee), to promote lactation; calmative for children; and for losing weight	++
*Juniperus oxycedrus* L., Cupressaceae	Juniper (cade)	Quatran	I	Fr- > Oil	M: hair treatment (dying mean)	+
*Laurus nobilis* L., Lauraceae	Bay laurel	Wrqa sidna musar	L and I	Le	F: seasoning for chicken and red meat dishes; M: dried leaves are burned, and vapors inhaled for treating fever, to clear throat and help sleep	++
*Lavandula* spp., Lamiaceae	Lavender	Khzama	L	Fl	M: tea used for lung problems, rheumatisms, stomach-ache, bladder problems, and constipation	++
*Lawsonia inermis* L., Lythraceae	Henna	Alhana	L	Le	M: paste used to color hair or decorate skin	+
*Lepidium sativum* L., Brassicaceae	Garden cress	Hab rchad	L	Se	M: to “warm the body” and to prevent colds	++
*Linum usitatissimum* L., Linaceae	Flax	Zeret kitan	L	Se	F: garnish for sweets and breads	+
*Malva sylvestris* L., Malvaceae	Mallow	Khobiza	L (G)	Le	M: tea for cold and inflammations of the digestive tract	++
*Mentha pulegium* L., Lamiaceae	Pennyroyal	Fliou	L		M: tea for sore throats, cold, and fever	+++
*Mentha spicata* L., Lamiaceae	Spearmint	Nene	L	Le	M: recreational and digestive tea	+++
*Mentha suaveolens* Ehrh., Lamiaceae	Apple mint	Marseta	L (G)		F: occasionally gathered from the roadside and consumed on bread; F/M: consumed with honey as a strengthening agent, for improving mental capabilities and against colds	+
*Myristica fragrans* Houtt., Myristicaceae	Nutmeg	Gouza	L	Se	F: spice for sweets	+
*Nigella sativa* L., Ranunculaceae	Nigella sativa	Al habba assawda	L	Se	F: garnish for sweets and breads; M: remedy for colds, cough, promoting lactation, and treating bone problems; seeds burned, the smoke released throughout the house, with windows open: considered able to counteract negative energies	++
*Olea europaea* L., Oleaceae	Olive	Zeytun	L	Le, Fr- > Oil	F/M: oil considered good for general health; M: leaves in tea for treating high blood pressure	++
*Origanum majorana* L., Lamiaceae	Marjoram	Mardadouch	L	Le	F/M: seasoning and digestive agent M: recreational tea	+
*Origanum syriacum* L. and *O. vulgare* L. Lamiaceae	Bible hyssop and Oregano	Zaatar	L and I	Leaves	F: herb for some savory dishes F/M: also considered useful in food as a digestive aid (carminative), for reliving menstrual pains, as an anti-fever agent, against stomach-aches, as an anti-diarrheal agent, and for losing weight	+++
*Papaver somniferum* L. Papaveraceae	Poppy	Budhur alkhashkhash	L	Se	F: garnish for sweets and breads	+
*Phoenix dactylifera* L., Arecaceae	Date	Tmar	L and I	Fr	F/M: consumed for providing strength and energy (“Muslims should eat 3, 5 or 7 dates every day, like the Prophet Mohammad”)	+++
*Pimpinella anisum* L., Apiaceae	Anise	Nafaa, Yassun, Habat hlewa	L	Fr	F: garnish for sweets and breads; M: tea considered good as a digestive and for losing weight (sometimes fruits ground with coffee)	+
*Piper nigrum* L., Piperaceae	Black pepper	Al folfol al aryad	L	Fr	F: *belbula* (dish made with wheat, milk, and pepper) F/M: seasoning as a digestive aid	+++
*Pistacia lentiscus* L., Anacardiaceae	Mastic	Maska al hurra	I	Re	F/M: as a natural chewing gum, for treating toothaches	++
*Punica granatum* L., Lythraceae	Pomegranate	Raman	L	Fr	F/M: consumed for general health (as one of the foods mentioned in the Quran it is thus believed curative)	+
*Rosa* spp., Rosaceae	Rose	Waradi	L	Fl	F: flavoring for sweets; F/M: considered an aphrodisiac	+
*Rosmarinus officinalis* L., Lamiaceae	Rosemary	Iklil al jabal, Azir	L	Le	M: tea for relieving sore throats and good for the general health	++
*Salvadora persica* L., Salvadoraceae	Tooothbrush tree	Miswak	L and I	Wo	M: oral hygiene, externally applied; in teas, for treating stomach problems and during menstruation	+++
*Salvia officinalis* L., Lamiaceae	Sage	Salmiya	L	Le	M: tea for relieving sore throats and heart problems; with lemon for treating stomach-aches	++
*Sesamum indicum* L., Pedaliaceae	Sesame	Assimssim, zanjlan	L	Se	F: garnish for sweets, breads, and savory dishes F/M: strengthening food	++
*Syzygium aromaticum* (L.) Merr. and L.M.Perry, Myrtaceae	Cloves	Al koronfol	L	Fb	F: cooking spice for savory and sweet dishes	+
*Thymus vulgaris* L., Lamiaceae	Thyme	Zaetar	L	Le	M: tea for treating stomach diseases	+
*Trigonella foenum-graecum* L., Fabaceae	Fenugreek	Al halba	L	Se	F: cooking spice for savory dishes (esp. cous cous, lentils, breads); M: tea as a digestive aid, an anti-fever agent, an appetite stimulant, and for promoting lactation; seeds, powdered and made into a paste with water, applied directly to skin for treating blemishes	++
*Zingiber officinale* Roscoe, Zingiberaceae	Ginger	Skinjbir	L	Ro	F: used in many savory dishes; F/M: in food it is considered able to treat colds and to have anti-inflammatory properties; M: used externally in massage oils; in tea with lemon for the prevention of colds and as an anti-fever agent	+++

*Ap* aerial parts, *Ba* bark, *Bu* bulbs, *Fb* flower buds, *Fl* flowers, *Fr* fruits, *Le* leaves, *Re* resin, *Ro* roots, *Se* seeds, *Sg* stigma, *St* stems, *Tu* tubers, *Wo* wood, *Wp* whole plant

+, mentioned by less than 10% of the study participants; ++, mentioned by 10–39% of the study participants; +++, mentioned by at least 40% of the study participants

**Fig. 2:**
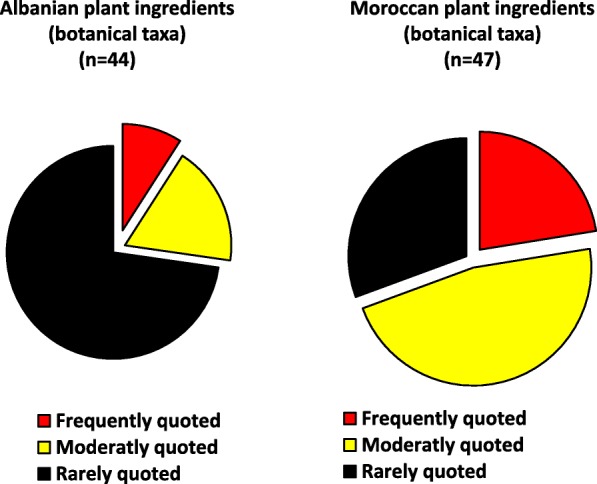
Quotation frequency of the home country plant ingredients used by Albanian and Moroccan migrants

**Fig. 3:**
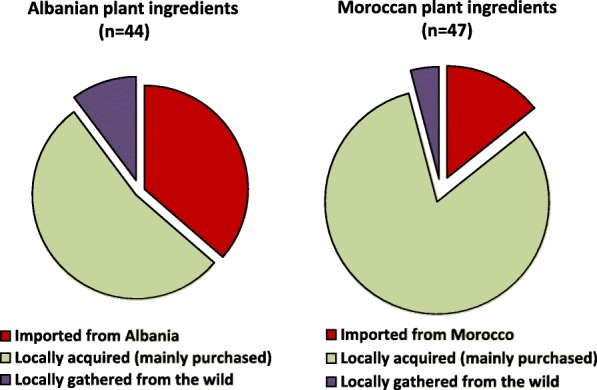
Ways of procurement of the home country plant ingredients used by Albanian and Moroccan migrants

**Fig. 4:**
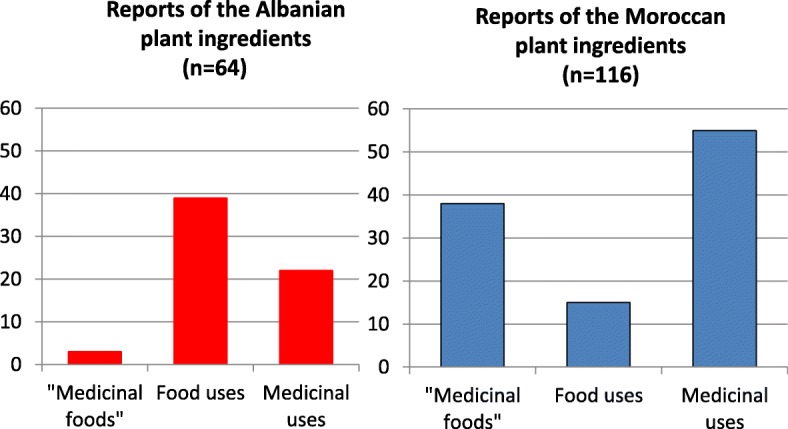
Domestic uses of the home country plant ingredients mentioned by Albanian and Moroccan migrants

While the number of plant ingredients (botanical taxa) was similar in the two communities (44 plant items among Albanians vs 47 plant items among Moroccans), the data diverged remarkably on the following three trajectories:

- frequency of quotation: while most of the home country plant ingredients were frequently or moderately mentioned by Moroccan migrants, the majority of those mentioned by Albanians were rarely mentioned as still in use in Italy (Fig. 2);
- ways through which the home country plant ingredients are acquired: while most of the ingredients used by Moroccans were purchased in local (ethnic) shops and markets, a considerable portion of the ingredients used by Albanians were informally “imported” during family visits from Albania (Fig. 3);
- quantitative and qualitative differences in the plant reports mentioned by the two communities: (a) the plant reports recorded in the domestic arena of Moroccans were very diverse and nearly doubled the reports recorded among Albanians and (b) most of the plant ingredients mentioned by Albanians are used only as food ingredients, whereas most ingredients mentioned by Moroccans represent “food-medicines” (Fig. 4).

### Eating Albanian and Moroccan in Italy: a different resilience of traditional botanical and gastronomic knowledge

The frequency of quotation of the home country plant ingredients (Fig. 2) clearly shows that while Moroccans in Bra and Turin often referred to most of them as being used in their domestic practices; Albanians mentioned them much more rarely. According to our open interviews, this is clearly related to the different attitudes that the two communities have towards their home country culinary traditions.

In fact, most of the Albanian migrants reported eating predominantly Italian food after moving to Italy and many of them claimed to be accustomed to it, having been exposed to Italian food even before migration, albeit on a very different scale (more than two thirds of all the Albanian study participants reported eating Italian food at least once or twice a week while they were still in Albania). The cultural, economic, and social links to Italy were celebrated by many Albanians as part of the “modernization” process, and thus, there was a period of rapid absorption of Italian food, culture, and ideals [54]. Additionally, a portion of our Catholic Albanian study participants were very proud to have been “Italianized,” due in part to intermarriage with Italians, while this positive attitude was not shown by Muslim Albanians, who still appreciated Italian cuisine. The frequency of cooking Italian meals at home among Albanian migrants was also directly related to time spent in Italy: according to our interviewees, the average number of Italian meals prepared at home increased from approx. 1.5 to 3 per week after 10 years of residence in Italy, whereas Albanian meals were prepared only once per week on average and remained constant while staying in the host country. The gastronomic familiarization with the host country cuisine was enhanced through experience and a deepening of the knowledge of Italian national and even regional dishes. When asked to list the Italian food they knew and liked, some Albanian respondents that had been in Italy more than 5 years also mentioned—apart from *lasagne*, *pizza*, *and* various Italian pork-based cured meats (*prosciutto* and *salame*)—“*agnolotti”* (Piedmontese ravioli) and “*trofie al pesto*” (Genoese home-made, short, twisted pasta dressed with basil pesto sauce). Those who had spent less time in Italy or migrated back more frequently typically listed only generic Italian dishes. This indicates that in the food arena, Albanian migrants did not seem willing to counteract the hosting community, defining themselves in opposition to the other migrant communities. This particular attitude also reverberates in the commercial data. Despite the large number of immigrants of Albanian origin, there are no Albanian ethnic shops in the entire Piedmont Region (42,000 migrants), and there are very few Albanian restaurants in Italy.

On the other hand, many Moroccan respondents claimed to mainly cook Moroccan dishes in Italy. Several participants noted that Italian dishes were often less time consuming to prepare than Moroccan dishes, and the few respondents who were used to preparing more Italian than Moroccan dishes affirmed that they did so for this reason. Open interviews also showed how religious beliefs play a crucial role among Moroccan respondents. In particular, they pointed out that eating meat slaughtered according to halal standards is a hallmark of their foodways. Most of the respondents claimed that this aspect is very important to them and that they wanted their children to follow this rule. These particular gastronomic practices resonate in the commercial data. In particular in Turin, as well as the other large cities of the region, such as Asti and Alessandria, Moroccan migrants manage a wide network of food shops, in particular butcher’s shops and groceries, which sell halal meat and imported goods from Morocco and other North African countries, as well as additional items of everyday use, such as sugar, coffee, and rice. Moreover, Moroccan migrants play an important role in the Italian street food scene because of the *kebab* shops. Although they originated in Turkey or, more exactly (for the rotating version), among the Turkish diaspora community in Germany, *kebab* shops have spread enormously in the last decade in Piedmont, mainly via Moroccan and Egyptian migrants [55], although they are very popular among young Italians as well. Indeed, the *kebab* is a transnational icon [56, 57], representing a westernized idea of Oriental cultures that has been transformed into a blend of different kinds of food and an object of the interaction [58]. In fact, in many *kebab* shops managed by Moroccans in Bra and Turin, it is possible to find not only *kebab*, but also pizza and other yeast-leavened products. This hybridization indicates that the *kebab* is not only part of the foodscape of young Italians [59], but also the lynchpin of a cultural integration process through gastronomy. Interestingly, however, this integration process was perceived to be somewhat one-sided. Many respondents in Bra felt that the local Italian community was not particularly interested in Moroccan culture. When asked why there were no Moroccan restaurants in town apart from the *kebab* shops, even with such a large, well-established Moroccan community, respondents reported that local Italians are not very open-minded about other food cultures and that there are not enough young people in the community who might take an interest in Moroccan restaurants.

### Albanian and Italian cuisines among Albanian migrants

Italian cuisine is well known in Albania given the country’s long-lasting historical ties to Italy. Many interviewed Albanians confirmed that they were familiar with Italian cuisine before coming to Italy, even if they very rarely tried it in Italian restaurants in Albania before migrating. One respondent reported:

“Due to geographic vicinity and the major influence of Italian language, television, music, culture in general in Albania, the gastronomic traditions have been mixed up for many years already, more evident after 1992. Currently, in Tirana there are many Italian restaurants run by Albanians and Italians, and also in our daily dishes pasta, pizza, and a few other ingredients are regularly present”. (Female, 43 years old).

The main differences between Italian and Albanian food were related to the greater diversity of Italian dishes, the presence of more meat in Albanian cuisine, and the distinction between “dry” food (Italian) and “wet” food (Albanian); for example, the greater presence of soups and yogurt-based dishes in the latter.

Another comment that was made many times was the perceived difference between heavy, butter-based, Albanian foods versus lighter, healthier, and easier to prepare Italian dishes. For example, one 48-year-old woman argued: “Albanian food is too heavy… I always receive so much pressure from my mom to eat it.”

All respondents described Italian cuisine in glowing terms, such as delicious, balanced, “synonymous with healthy eating,” and “the best in the world.” All participants attributed to Italian food a complexity of flavors and a variety of ingredients unseen in Albanian cuisine, which they instead viewed as greasy, salty, and rich in animal fats. While almost all migrants expressed some degree of nostalgia for Albanian food, or recognized it as a formative part of their identity in connection to home and family, every respondent labeled Albanian food as unhealthy and conservative. Many respondents acknowledged that Albania’s energy-dense cuisine was the result of an *identity-driving* agro-pastoral and especially pastoral past. As such, some Albanians characterized their traditional foods as something anachronistic, closed-minded, and unrelated to their modern, “westernized” lives.

Albanian participants expressed an ambivalent relationship with their own cuisine, as it is considered a factor of cultural identity but also of poor quality, especially in comparison to the Italian one. Since the first Albanian migration waves, Italian cuisine has gained more international prestige, which could also be affecting the attitudes that Albanian study participants express. Italian food was perceived by participants as synonymous with emotion, experience, and adventure, perhaps driving their interest.

### Moroccan and Italian cuisines among Moroccan migrants

Moroccan migrants had a different perception of the relationship between Italian and Moroccan cuisine. Firstly, the Moroccan respondents tended to associate their traditional cuisine with the South Italian one only. One 50-year-old female respondent stated, for example: “Our cuisine is more similar to the South Italian one because our soil is warm.” This association may be due to the fact that both Moroccan and South Italian cuisines belong to the Mediterranean gastronomic culture, while the NW Italian cuisine does not.

Some declared that both these cuisines are very diverse and there are few differences between them as they use the same ingredients, although Moroccan cuisine was characterized by more intense flavors and more spices.

A few respondents emphasized the fact that, in their opinion, products in Morocco are fresher and more flavorful than Italian ones. One interviewee (48-year-old female) attributed this to differences in the climate of the two countries, while another study participant (62-year-old male) pointed out differences in gastronomic habits regarding the way of eating and the time for having meals:

“In Morocco breakfast is generally salty with bread, cheese, olive oil, butter, and sometimes honey and jam. Occasionally we have coffee during the afternoon as a snack. We never drink coffee at breakfast. In Morocco, we eat everything on the same plate, called tajin. This is because sharing food means the union and love of our family”. (male, 62 years old).

The Moroccan community claimed the goodness of its gastronomic identity, although it recognized a proximity to the culinary traditions of Southern Italy, for geographical and cultural reasons. In this case, food proved to be a fundamental element of cultural identity and pride.

It would have been interesting to see how these perceptions among our Moroccan study participants may have been influenced by the growth of Islamophobia in Northern Italy, a process that has been ongoing for a decade.

### Gender relations and food within migrant households

A study [60] from two decades ago analyzed how the migration process has changed the relationship between men and women, bringing new values to the home country along with the acceptance of a more gender-balanced worldview [61] through an empowerment process and an increase in the decision making power of women, especially when there is a woman (a daughter or a sister) who sends remittances to the home country [62].

Nearly all respondents mentioned mothers, grandmothers, or aunts as being responsible for preparing meals in Albania. According to our interviews, in Albania, food preparation, the gathering of ingredients, and general management of the kitchen is completely a woman’s domain. Albanian males living in Albania traditionally rarely learned to cook. However, this scenario changed upon moving to Italy. With few exceptions, interviewed Albanian men stated that they can also cook for themselves. This could be because men were usually the first ones to migrate. On the other hand, when the family was reunified, cooking again became a domain mostly dominated by women and the patriarch of the family, instead, only cooked sporadically (generally roasting meat). Albanian men saw their role in the reunified families as the main *home-country knowledge holders*, maintaining contact with family members in Albania, and following Albanian-language news and media.

In the case of Moroccan migrants, however, it appeared that such a change in gender roles related to food never occurred, and cooking remains women’s work. Even in interviews where male members of the household were present, female respondents (primarily mothers and grandmothers, but also daughters) did most of the talking, and husbands tended to defer to their wives when asked about the details of the uses, both culinary and medicinal, of the various mentioned ingredients. In fact, the Moroccan interviewees considered women as the authorities on food and medicine within the domestic arena and responsible for preparing food and administering care, regardless of their employment status. Even if second-generation adolescents absorbed and reflected Italian cultural values in the public sphere, in the domestic arena, Moroccan identity and cuisine was cultivated.

### Traveling plants: informal exchanges with the home country

Figure 3 shows the ways through which the home country plant ingredients were acquired: while most of the ingredients used by Moroccans were purchased in local (ethnic) shops and markets, a considerable portion of the ingredients used by Albanians were informally “imported” during family visits from Albania.

This particular configuration of food demand can be explained, on the one hand, by the different practicability of having regular, frequent exchanges between Piedmont and Albania (with a number of low-cost flights departing every day to Tirana), and, on the other hand, by the availability of traditional ingredients used in Moroccan cuisine in the wide network of shops run by Moroccan and Northern African migrants. However, this materialistic interpretation risks overlooking the relational and affective dimension that the interviews suggested were crucial for the Albanian respondents. In fact, the informal exchange of food appeared to play an important role in strengthening family and kinship relations between Albanian émigrés and their relatives remaining in the home country.

One 36-year-old woman stated:

“One month ago, my mom brought from Albania some herbal teas for me in Italy. She also brought some homemade cow cheese. I felt so much pleasure, I love cheese. Also, small tomatoes, peppers, and chilies in brine; we used to eat a lot of pickles in Albania. And cigarettes of course!”

According to our respondents, the role of relatives sending food abroad was important in keeping ties with their country of origin, especially for those migrants who were still suffering from family or community separation. Another very important identity-linked product that Albanians generally import through compatriots is *raki*, a home-made alcoholic drink obtained from the distillation of fermented fruits, often grapes, but also plums, cherry plums, Cornelian cherries, and mulberries. This was especially true for the Catholic portion of the Albanian migrant community, whose families originated in Northern Albania. Additionally, wild dried herbs (such as dried flowering aerial parts of wild oregano, mountain tea, and wild sage—*Origanum, Sideritis,* and *Salvia* spp., respectively), jars of home-made lacto-fermented pickled vegetables, and dairy products could be often found in the luggage of visiting family, as these ingredients are not available in Italian markets or their quality is considered to be superior. The latter reason was particularly stressed for home-made, white *feta-*like cheese (*diath*) and clarified butter (*tylënë*), often informally brought from Albania to Italy, as these are perceived as having been produced in a natural environment in the home country that is considered pristine and extraordinarily “clean.”

There was a contrasting situation with respect to the households of Moroccan migrants. Although a strong network exists among Moroccans in Piedmont, as well as between migrants and their families back in Morocco, members of this community have ready access to most, if not all, of the ingredients that they consider important for their cuisine and for traditional home remedies. Most of the Moroccan respondents claimed to be able to find most of the ingredients for Moroccan dishes and home remedies in Moroccan shops or in the market of Porta Palazzo (one of the largest open-air markets in Europe) in Turin. Our study participants said that they can find many vegetables that are cultivated in Morocco and then imported to Italy. Moreover, in the Porta Palazzo area, there are several ethnic shops, halal butcheries, and a bazaar managed by North African migrants where many Moroccan ingredients are readily available. The Moroccans we interviewed in Bra stated that they were generally able to find all the ingredients (culinary and medicinal) that they needed or desired within the town of Bra (in four shops in particular, two of which are owned and operated by Moroccans) or in Turin. As argued in [23], choosing to eat the traditional food sold by co-nationals is a way to affirm identity, help self-construction [63], and provide a sense of belonging to a community in contrast to the others.

### The loss of foraging knowledge

In both the Albanian and Moroccan samples, the foraging of wild plants was seldom reported. Only three Albanians admitted to foraging only very sporadically in spring in order to obtain wild vegetables which are crucial ingredients for preparing specific salty pies (*byrek*), while only one Moroccan respondent reported having gathered wild herbs in Piedmont.

However, most of our study participants stated that foraging was not something they would have done even before coming to Italy as these practices stopped during their parents’ generation.

One 53-year-old Moroccan woman justified the loss of traditional gastronomic knowledge in this way: “We don’t forage here because even the same plants [that we would find in Morocco] might taste different here [in Italy].”

### Differences in the plant reports by Albanian and Moroccan migrants

Figure 4 shows how the plant reports mentioned by Moroccans is double that of Albanians. This may again be linked to the different resilience of folk gastronomic and herbal knowledge and practices.

However, the different importance of herbal teas (more diverse uses among Moroccans) needs to be further studied. Home-made herbal teas in Moroccan and Albanian migrants’ households are not just beverages, they also play an important recreational and social role and can be considered a sort of *cultural marker* as well, if compared with the non-existent tradition of drinking tea in Italian food culture.

In the case of Moroccan migrants, a botanical cultural marker is spearmint; mint tea is served hot in Moroccan houses any time of the day and is a symbol of conviviality, friendship, and brotherhood, and is a ritual aspect of Moroccan culture still actively practiced in Piedmont.

One respondent also reported that spearmint tea is often accompanied by different kinds of bread. During the winter, *sheeba* (*Artemisia arborescens*) and pennyroyal (*Mentha pulegium*) are often added to tea—either in addition to or as a substitute for mint—to prevent flu and the common cold. Unsurprisingly, these two herbs came up more frequently in the interviews we conducted during the winter months.

According to several interviewed Moroccans, rosemary, sage, and lemon verbena may also be added to tea, either as preventative medicines or in response to various common ailments. All the Moroccans we met reported using both home country herbal medicines and Western biomedicines. Most of them preferred to use herbal remedies for common ailments in order to avoid the use of conventional painkillers (which “weigh the body down”) but would readily use conventional medicine if more serious health issues arose. Although some young respondents claimed to prefer using conventional medicine in all cases, they still consumed different traditional Moroccan herbal teas and agreed that these teas have positive effects on health.

Albanian respondents also reported iconic teas: *çaj mali* (literally “mountain tea”). This name refers to two main species, both categorized by the same folk term. In the North of the country (the Ghegh dialect speaking part) and in the migrant families that came from that area to Italy, dried flowering tops of *Origanum vulgare* are used, while in the South of the country and among (Tosk dialect speaking) South Albanians in Piedmont, the same tea is prepared using the dried aerial parts of *Sideritis* spp.; *çaj mali* plays a crucial role both as a recreational tea and as a panacea.

However, the most notable differences our study highlighted concerned the reports of “medicinal foods” (those ingredients indicated with F/M in the tables), which were prominent among Moroccans and quite irrelevant among Albanians.

In fact, Moroccans mentioned the use of several seasoning ingredients as home medicines, while among Albanians only *salep* (powdered dried wild orchids tubercles) and potato soup were mentioned.

This interesting finding is related to a greater use of aromatic plants and spices in Moroccan cuisine, which in past centuries was spread all over the Near East and Northern Africa by Arabs and whose “double nature” as both food and medicine is well-known in the literature. Moreover, this could also be linked to the fact that Moroccan émigrés faced difficulties in gaining access to the public health centers in Italy, which may have shaped a more resilient custom of preparing home-made “food medicines.” This may be related in turn to a perceived stronger cultural distance between Moroccans and their Italian neighbors, considering both linguistic barriers (most of the Albanian interviewees were perfectly fluent in Italian, which was not the case for first-generation Moroccan migrants) and cultural norms.

## Discussion

### Gastronomic deculturation, enculturation, or assimilation?

Migrant foodways and their adaptation to the Italian context reveal important information concerning the cultural adaptation strategies adopted by Albanian and Moroccan migrants in approaching the host environment. While acculturation is the result of two simultaneous processes (one involving deculturation from the original culture, and the other involving enculturation towards the adopted culture [14, 64]), the Albanian case study presented in this work shows much more than mere acculturation, since (as of yet) there is no public sphere in which Albanians can express their identity and share their food cultural capital (no ethnic enclave of Albanian restaurants, markets, and shops to reinforce this home-crafted culture exists in the study area). As such, Albanian-Italian families have thus far split their cultural identities into two, via a sort of mimesis, creating a public, Italianized self while safeguarding the private Albanian self to produce a kind of “acculturation without assimilation” [65]. In this context, eating Italian among Albanian migrants means something very different than eating the same foods they had been exposed to in Albania. To them, Italian cuisine is much more than just food recipes and ingredients: it is a way of life, a whole sphere of knowledge, history, and relationships, as well as etiquette and taboos. Albanian migrants described learning to “eat and be Italian” not only as a process of observing and mimicking a series of culinary processes and gestures, but also as a process of learning to “talk around food” like Italians, cataloging a vast store of gastronomical knowledge. For example, a 45-year-old Albanian woman stated:

“Albanians eat to live. Italians live to eat. I remember when I first came here, I couldn't believe that people just talked about food all the time! At school, work, in the streets... It wasn't until I came to Italy that I began to realize the importance of food.”

Another important aspect concerns the memorial and evocative function of food. When asked what Albanian cuisine represents to them, almost all the interviewees stated that it was a tool to go back to their origin, their childhood, and their national identity. One 32-year-old Albanian woman reported: “Sometimes I try to cook something traditionally Albanian because it reminds me of my grandmother but it’s difficult… I like it because it is something connected to memories, my mother cooking at home.”

At the same time, most Albanian interviewees primarily cooked Italian food, as mentioned earlier, because, as the previous informant pointed out: “Italian food is the present, it is the integration into a new culture.” In the absence of strong Albanian cultural institutions in Piedmont (such as a church/mosque or cultural center), the “home”/family has become the formative space in which Albanian migrants can negotiate their dual identities.

There were no noticeable differences in diet or acculturation between our Albanian study participants—of which half were Muslim and half Catholic. They tended to support the thesis that religious affiliation is irrelevant to food customs, suggesting that religion is “referred to in terms of family tradition rather than faith”, as pointed out in other studies [66–68]. This view is linked to both the particular historical patterns of the diffusion of Islam in Albania (with a crucial role played by the Sufi dervish order of Bektashis [69]) and the long-lasting Communist period, which contributed to the spread of irreligion (today, more than one third of the entire population of Albania seems to be non-religious, according to a 2011 survey [58]). Consequentially, Albanians also feel integrated by eating mainly Italian food, and consider that gastronomy provides a strong sense of belonging to the host country, more than other social and cultural spheres.

The case of Moroccan migrants was rather different and, in some ways, quite the opposite. Moroccan migrants in our study, and more generally in Europe, often experience difficulties in the acculturation process [62]. Many Muslim migrants to Italy endured a complex transition experience, characterized by high levels of perceived discrimination mainly based on their religious identification and as a result of the post-September 11th climate of fear as well [68]. Among these migrants, the home was the place for the perpetuation of language and traditions, and Moroccans were consciously aware of this fact; one 34-year-old Moroccan woman stated: “I cook Moroccan food almost every day, to teach my young children.” Study participants claimed to cook mainly Moroccan dishes with ingredients imported, if possible, from Morocco. Indeed, a 54-year-old man claimed: “I think Italian culture impacted negatively on us because we disregarded our traditions.” This statement highlights a reluctance to deculturate, as well as the difficulty in finding a balance between the two cultures. Religion was strongly related to this reluctance. In fact, Moroccan respondents seemed to seek, in religious faith, a way to find their own identities, and the halal butchers and North African bazaars represented places in which to reassert their identities. However, many of our Moroccan respondents agreed that, despite the difficulties in the integration process, food can bridge the cultural divide. One Moroccan man (32 years old) recalled: “When my colleagues got to know where I was from, they wanted to taste cous-cous.” Later, they asked him to provide them with some Moroccan delicacies and he concluded: “This is the proof that food culture has helped me in the integration process”. This polyvalent role of food also contributes to new relationships outside the original ethnic group. Another Moroccan study participant (34 years old) reported an interesting case of gastronomic syncretism, a way of combining Italian and Moroccan food ingredients and identities. He stated: “My mother started using ingredients of both cultures when cooking. For example, she adds fresh ginger and cumin into the pasta tomato sauce, or she adds rosemary in the tajin meat.”

The attitude of the Moroccan community seemed to be influenced by cultural and religious dynamics, which claimed Islamic identity in all aspects of social life. In this sense, we have already mentioned how food represents a strategic element of the individual and collective identity. In everyday practice, however, this cultural resistance seems almost to dissolve in the shops selling kebabs and in the multi-ethnic recipes adopted.

Food knowledge adaptation is known to be partly the result of a complex cultural negotiation between the host and home cultures and environments of a migrant population [19]. This negotiation tends to happen within a complex socio-cultural spectrum influenced by numerous variables in the host environment. In this respect, analyzing Albanian and Moroccan foodways, we observed how the two migrant groups followed different adaptation strategies for shaping their cultural identities within the host environment.

A large portion of the differences are linked to different methods of procurement of home country gastronomic botanical ingredients, to different transnational informal exchanges between Italy and the home country, to the presence of markets and ethnic shops in Italy selling these items, and to the different degree of difficulty in accessing public health services. However, the observed divergences are also clearly linked to a different mirroring of what the representation of *Albanianness/Moroccanness* vs. *Italianness* means in daily life.

Albanian migrants tended towards acculturation to the Italian mainstream and social mimicry as well, undergoing a process that King and Mai labeled the “Albanian assimilation paradox” [56]. Albanians used social mimesis especially in the first years of life in Italy as a cultural survival strategy: literature as well as personal observation attest to the phenomenon of Albanian migrants pretending to be Italians, especially one to two decades ago, both in Italy and in other Western countries [69 and several personal observations], possibly also in order to overcome the social stigma they have to go through, while employing food to navigate a dual identity across public and private spheres.

Using food as a way of approaching Italian identity, the Albanian community has succeeded in integrating, both culturally and socially, while the geographical proximity of the two countries favors frequent exchanges, making the relationship between Italian society and Albanian migrants more solid and firm.

On the other hand, Moroccan migrants tend to approach the new food culture more slowly, trying to preserve their traditions as much as possible, thus experiencing a kind of *food cultural resistance*. This could also be due to their religious and cultural sphere, which influences the way they eat, its representation, and the consequent process of integration through food culture.

In the face of these different adaptation strategies, the research finds a fundamental difference in the trajectories of transformation of traditional botanical knowledge. It is commonly assumed that, as communities relocate, traditional domestic food and medical knowledge is usually lost through acculturation [14, 21]. However, one study revealed that in a heterogeneous urban community, traditional knowledge regarding medicinal plants was retained and even rejuvenated with additional popular knowledge [13]: this was perhaps possible as urban centers, in contrast to rural areas, tend to host a greater diversity of ethnic markets selling traditional foods and medicinal plants and provide migrant communities with comparatively easy access to both items from their home country and those from the home countries of other migrant and autochthonous neighbors.

In a previous study, we showed how food and medical habits are a very good proxy of the extent to which a migrant group has assimilated the host culture [16], and while a migrant community often has difficulties in its political, economic, and social expression, food is an easy way to confirm and consolidate identity through the simple act of choosing specific food ingredients, preparing certain dishes, and eating [30].

Further studies will be required to show the evolution of these strategies, and if the Moroccan, or, more presumably, the Albanian pathway develops into a proper cultural assimilation, in which traditional knowledge is eroded more and more in the process of adaptation and home country plant ingredients are forgotten or exceptionally gathered through informal exchanges. It would be especially interesting in future research to observe this phenomenon among the various Albanian diasporas in the world (most notably in the USA and Greece), and it will be crucial to do so in order to assess the potential effect of the emerging *Albanian nouvelle cuisine*, which is finding its ascendant momentum at this time, in part through “gastronomic expeditions” organized by avant-garde Albanian chefs in search of the “hidden treasures of the Albanian gastronomic heritage” [70–72].

## Conclusions

This research investigated the adaptation processes of folk plant knowledge among Albanian and Moroccan migrant communities living in Turin and Bra, NW Italy. Focusing on the gastronomic knowledge, practices, and believes of first- and second-generation migrants, the conducted research pointed out the differences between the folk plant knowledge of the two communities, which are linked to different methods of procurement of home country gastronomic botanical ingredients, different transnational informal exchanges that exist between Italy and the migrants’ home countries, a different presence of markets and ethnic shops in Italy selling these items, and a different degree of difficulty in accessing public health services.

The analysis of the different trajectories of change that affect folk plant knowledge contributes towards a better understanding of the links between the changes of folk knowledge and the cultural adaptation strategies followed by migrant communities. The study suggests the need for further interdisciplinary research investigating the complexity of folk gastronomic continuity and change, especially under a transcultural point of view and among other Albanian and Moroccan diasporas as well as in Albania and Morocco.
